# NMR Study of Intercalates and Grafted Organic Derivatives of H_2_La_2_Ti_3_O_10_

**DOI:** 10.3390/molecules25225229

**Published:** 2020-11-10

**Authors:** Marina G. Shelyapina, Oleg I. Silyukov, Irina P. Lushpinskaia, Sergey A. Kurnosenko, Anton S. Mazur, Ilya G. Shenderovich, Irina A. Zvereva

**Affiliations:** 1 Department of Nuclear Physics Research Methods, Saint-Petersburg State University, 7/9 Universitetskaya nab., 199034 St. Petersburg, Russia; ira.lushpinskaya.94@mail.ru; 2 Institute of Chemistry, Saint-Petersburg State University, 7/9 Universitetskaya nab., 199034 St. Petersburg, Russia; silyukov@spbu.ru (O.I.S.); st040572@student.spbu.ru (S.A.K.); irina.zvereva@spbu.ru (I.A.Z.); 3 Magnetic Resonance Research Center, Saint-Petersburg State University, 7/9 Universitetskaya nab., 199034 St. Petersburg, Russia; a.mazur@spbu.ru; 4 Faculty of Chemistry and Pharmacy, University of Regensburg, Universitätsstr. 31, 93040 Regensburg, Germany; Ilya.Shenderovich@chemie.uni-regensburg.de

**Keywords:** layered perovskite-like oxides, organic-inorganic hybrid, intercalation, grafting, NMR

## Abstract

The protonated perovskite-like titanate H_2_La_2_Ti_3_O_10_ has been used to produce organic-inorganic hybrids with simple organic molecules: methylamine, methanol, monoethanolamine, and *n*-butylamine. The optimal pathways for the preparation of such hybrids are summarized. Solid-state NMR, combined with thermal analysis, Raman, and IR spectroscopy, has been applied to determine the bonding type in the obtained organic-inorganic hybrids. It has been found that, in the methanolic hybrid, the organic residues are covalently bound to the inorganic matrix. In contrast, in the methylamine and *n*-butylamine hybrids, the organic molecules are intercalated into the inorganic matrix in cationic forms. The structure of the monoethanolamine hybrid is composite and includes both the covalently bound and intercalated organic species.

## 1. Introduction

Organic-inorganic hybrid composites are now widely used in the development of innovative functional materials for photovoltaics, photocatalysis, optoelectronics, and pharmaceutics [1,2]. One of the ways to obtain such hybrid materials is the incorporation of organic molecules into an inorganic matrix; in particular, this approach is often implemented in the case of layered inorganic compounds [3]. Among other inorganic matrices, layered perovskite-like oxides look like one of the most promising, as they themselves exhibit photocatalytic activity [4] that can be enhanced through various modifications [5]. Ion-exchangeable-layered perovskite-like oxides M*_m_*(A*_n_*_−1_B*_n_*O_3*n*+1_) are solid crystalline substances possessing a block-type structure in which perovskite slabs (A*_n_*_−1_B*_n_*O_3*n*__+1_) with the thickness of *n* BO_6_ octahedra alternate with interlayer spaces containing alkali cations M [6]. Such perovskite-like oxides demonstrate relatively high chemical reactivity in ion exchange [7,8], intercalation [9,10], and exfoliation processes [11,12,13,14]; some of them have practically significant photocatalytic [5,15,16,17,18,19,20,21,22], electrophysical [23,24], and luminescent [25] properties.

A treatment of ion-exchangeable perovskite-like oxides with acids cause a replacement of interlayer alkali cations with protons, giving so-called protonated forms. In this form, these perovskite-like oxides are able to react with some organic compounds, forming inorganic-organic hybrids—substances consisting of chemically bonded inorganic and organic parts, in which the inorganic one serves as a spatial frame [26]. According to the type of bonding between the inorganic and organic parts, there are two ways of hybrid formation: intercalation and grafting [27]. Intercalation is a reversible noncovalent introduction of organic bases (primarily amines) into the interlayer space by the acid-base or ion-exchange mechanism [28]. Covalent inorganic-organic hybrids can be produced by grafting reactions—the condensation of the protonated forms and appropriate organic substances (alcohols [29], alkoxysilanes [30], carbohydrates [31], carboxylic, and organophosphorus acids [32]), which is accompanied by the formation of covalent bonds B–O–C (B = Ti, Nb, Ta, etc.). These hybrids are of high interest for study due to the possibility of combining and fine-tuning useful properties of the inorganic and organic parts in one material [33,34]. The properties of hybrid materials are governed beside others by the type of chemical bonding between the organic and inorganic parts. Intercalated hybrids not only combine the physicochemical properties of both inorganic and organic parts but may exhibit synergetic behaviors afforded by both moieties, making possible the development of novel physicochemical devices [35,36]. Moreover, they can be used as an intermediate step for the synthesis of covalent hydrides [37]. Grafted derivatives have higher thermal and chemical stability, which is required for practical applications in an aggressive environment or further chemical modification [38]. In addition, by their exfoliation, such compounds can be used to obtain perovskite monolayers with a surface modified by organic molecules. The latter moderates both the aggregation stability of these monolayers in various solutions and their physicochemical properties [39,40,41].

Layered perovskite-like titanates H_2_Ln_2_Ti_3_O_10_ (Ln = La or lanthanide) are the protonated forms of the Ruddlesden-Popper phases A_2_Ln_2_Ti_3_O_10_ with the thickness of the perovskite layer *n* = 3. Since TiO_6_ octahedra in these compounds have unequal B–O distances due to the different local surroundings of oxygen anions, interlayer alkali cations are sufficiently mobile. These titanates demonstrate ionic conductivity [42] and pronounced ion exchange properties [43,44].

Titanates H_2_La_2_Ti_3_O_10_ are known to be able to form some inorganic-organic derivatives, with *n*-alkylamines [45,46] and *n*-alcohols [28] possessing the chain lengths of three carbon atoms and higher. At the same time, there are no detailed data on hybrids with the simplest representatives of amines and alcohols. Besides this, the literature does not cover the issue of obtaining hybrids with amino alcohols—bifunctional organic substances potentially capable of the simultaneous formation of both noncovalent and covalent bonds with perovskite slabs. All types of hybrids may have their own application fields, and it is important to monitor the product yield while developing synthetic methods.

Nuclear magnetic resonance (NMR) is a convenient tool for such monitoring due to its sensitivity to the electron density distribution in the vicinity of the resonating nucleus and, hence, to the type of a chemical bond [47]. Moreover, this technique provides information on the dynamics of intercalated species [10,48,49,50] and is successfully applied to study organic-inorganic-layered materials [10,48,51,52,53].

In this study, we used multinuclear NMR to characterize organic-inorganic hybrids based on the layered perovskite-like titanate H_2_La_2_Ti_3_O_10_ and lower amines (methylamine), alcohols (methanol), and amino alcohols (monoethanolamine), as well as *n*-butylamine. The NMR investigations were supported by the structural, elemental, morphological, and thermal analyses of the studied organic-inorganic hybrids.

## 2. Results and Discussion

The organic-inorganic hybrids were prepared according to the method described in Section 3. Further, in the text, tables, and figures, the studied materials will be denoted as follows: the layered protonated perovskite, H_2_La_2_Ti_3_O_10_·*x*H_2_O-HLT_3_, the methylamine derivative, H_2_La_2_Ti_3_O_10_ × MeNH_2_-HLT_3_ × MeNH_2_, the *n*-butylamine derivative, H_2_La_2_Ti_3_O_10_ × BuNH_2_-HLT_3_ × BuNH_2_, the methanol derivative, H_2_La_2_Ti_3_O_10_ × MeOH-HLT_3_ × MeOH, and the monoethanolamine hybrid, H_2_La_2_Ti_3_O_10_ × MEA-HLT_3_ × MEA.

### 2.1. XRD Analysis

**Figure 1:**
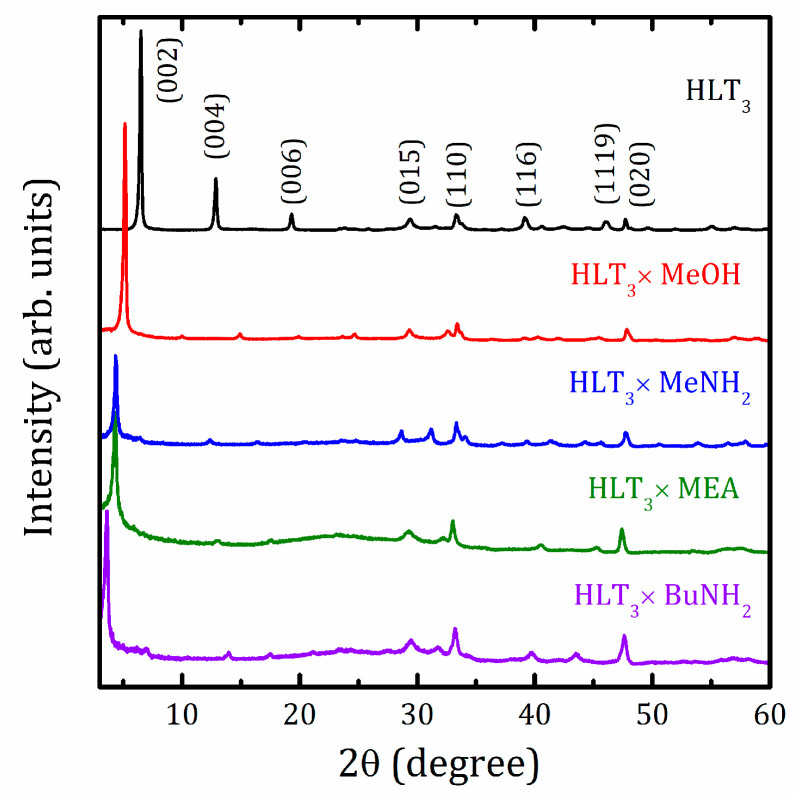
X-ray-diffraction (XRD) patterns of the initial H_2_La_2_Ti_3_O_10_·*x*H_2_O (HLT_3_), and organic-inorganic HLT_3_ × MeOH, HLT_3_ × MeNH_2_, HLT_3_ × MEA, and HLT_3_ × BuNH_2_ compounds.

To prove the formation of organic-inorganic hybrids, the materials synthesized were characterized by X-ray-diffraction (XRD) at each synthetic stage. Figure 1 demonstrates the XRD patterns of the initial protonated form and single-phase organic-inorganic compounds obtained under optimized conditions. More information can be found in the Supplementary Materials: Table S1 and Figures S1–S5. In all cases, the reflections observed are amenable to indexing in the tetragonal system. Formation of the hybrids is accompanied by a noticeable increase in the c lattice parameter, which is known to be directly related to their interlayer distance *d*, whereas the lattice parameter is seen to stay almost unchanged (Table 1). The observed increase in the interlayer distance is generally consistent with the molecule sizes of methylamine (~3 Å), *n*-butylamine (~6.7 Å), and a methyl group (~2 Å) of methanol (assuming grafting) if we take into account the additional expansion caused by intercalated water molecules and possible bilayer arrangement. At the same time, the interlayer distance of the monoethanolamine hybrid is consistent with the single monoethanolamine molecule size (5.3 Å), similar to what was observed in the case of the HLnTiO_4_ (Ln = La and Nd) hybrids studied earlier [54].

### 2.2. Raman and IR Spectroscopy

**Figure 2:**
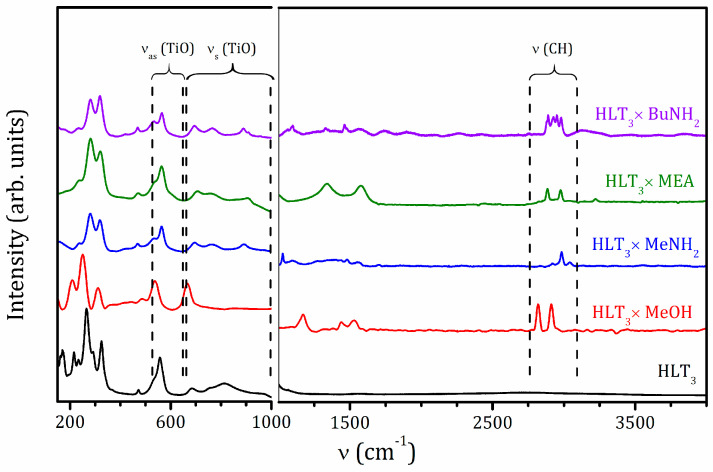
Raman spectra of the initial HLT_3_ and organic-inorganic HLT_3_ × MeOH, HLT_3_ × MeNH_2_, HLT_3_ × MEA, and HLT_3_ × BuNH_2_ compounds.

Raman spectra of the initial protonated form and obtained hybrid compounds are shown in Figure 2. The fact of the formation of the hybrid is confirmed by the appearance of characteristic bands, which are not observed in the spectra of the precursor—stretching of C–H (2800–3000 cm^−1^) and N–H (3225 cm^−1^) bonds, as well as latitudinal vibrations of methyl (1480 cm^−1^), methylene (1460 cm^−1^), amino (1560–1580 cm^−1^), and C–O–H (1340 cm^−1^) fragments. In the low-frequency region (<350 cm^−1^), which contains complex vibrations of the interlayer components linked to the oxygen of TiO_6_ octahedra, the main changes are related to the symmetric stretching mode (ν_s_) of the axial Ti–O bonds (810 cm^−1^ for the protonated form), which splits into two bands (765 and 895 cm^−1^) during amine intercalation, which points at the existence of two types of octahedra with unequal axial Ti–O distances. Bands of asymmetric stretching mode (ν_as_) of the closest to the interlayer space TiO_6_ octahedra (500–600 cm^−1^) and vibrations of the central weakly distorted octahedra (470 and 685 cm^−1^) do not experience noticeable changes during hybrids formation, indicating the preservation of the perovskite structure [55,56].

For the methanolic hybrid HLT_3_ × MeOH, the band at 810 cm^−1^, which is typical of the protonated form, does not exist; instead, a new band at 665 cm^−1^ appears. This may be related to the formation of covalent Ti–O–C bonds. Additionally, new bands at about 485 cm^−1^ emerge, and the bands at 560–570 cm^−1^, which can be referred to as the asymmetric stretching mode of TiO_6_ octahedra, shift to 540–550 cm^−1^, suggesting the noticeable influence of the methoxy groups on the perovskite octahedra. Moreover, bands associated with the C–O–H fragment vibrations that present in the spectra of the pure methanol, are not observed. These facts indicate that the interlayer space of the methanolic hybrid does actually contain not the molecular methanol but its methoxy groups covalently bound to the inorganic frame. It was previously shown that, depending on the synthesis conditions, amino alcohols can be incorporated with or without the formation of a covalent bond [37]. In the case of the monoethanolamine derivative, HLT_3_ × MEA, the observed vibrations of the C–O–H fragment (1340 cm^−1^) indicate the partial or complete presence of the interlayer monoethanolamine in a nongrafted form.

The Infrared (IR) absorption spectra of the obtained hybrids can be found in Supplementary Materials Figure S6. They are in fair agreement with the Raman data and, also, demonstrate the latitudinal vibrations of water (1630 cm^−1^) and stretching of its O–H fragments (wide band at 2800–3500 cm^−1^), suggesting the presence of intercalated water in the samples.

### 2.3. Thermal Analysis

**Figure 3:**
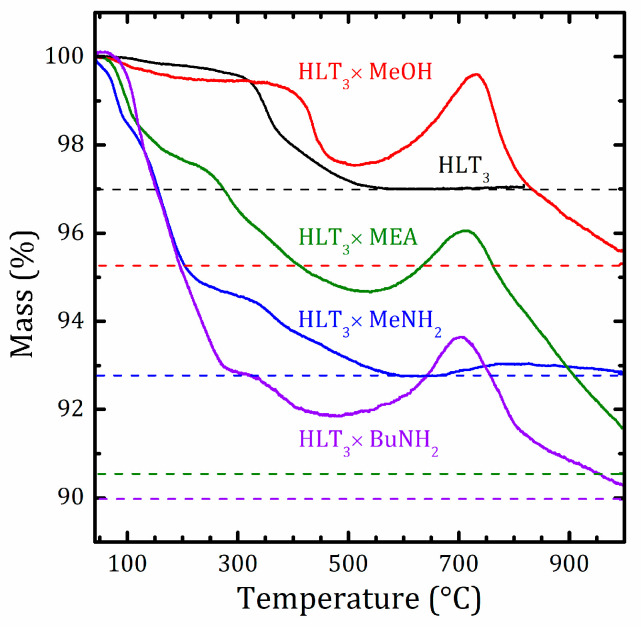
Thermal gravimetric (TG) curves for the initial HLT_3_ and organic-inorganic HLT_3_ × MeOH, HLT_3_ × MeNH_2_, HLT_3_ × MEA, and HLT_3_ × BuNH_2_ compounds. Masses after the final isothermal step are shown by dashed lines.

The degree of hydration and the temperatures corresponding to the loss of water and organic molecules were determined by thermogravimetric analysis with simultaneous mass spectrometric identification of the released gases (STA-MS). The thermal gravimetric (TG) curves are shown in Figure 3. The more detailed information on the STA-MS analysis can be found in Supplementary Materials Figures S7–S10. The thermal degradation of the methylamine hybrid HLT_3_ × MeNH_2_ in the oxidizing atmosphere proceeds as deintercalation. The evacuation of the intercalated water takes place at temperatures above 50 °C, followed by methylamine evacuation above 100 °C, giving the deintercalated protonated form, which decomposes at 300–500 °C. HLT_3_ × BuNH_2_ undergoes thermal degradation mainly in the same way as HLT_3_ × MeNH_2_. The only difference is observed at temperatures above 500 °C; in HLT_3_ × BuNH_2_, there is a noticeable mass gain stage, obviously associated with oxidation, which is replaced by the further mass loss stage related to the carbon dioxide release; see Supplementary Materials Figures S7 and S8. This suggests the existence of some remaining carbon-containing substances in the sample after the first stage of the decomposition. The same mass gain stages are also observed for the HLT_3_ × MeOH and HLT_3_ × MEA compounds.

According to the STA-MS data (see Supplementary Materials Figure S9), the organic part of HLT_3_ × MeOH does not decompose up to relatively high temperatures (approximately 300 °C), and the deintercalation of molecular methanol does not take place. This confirms the covalent nature of methanol binding. The HLT_3_ × MEA hybrid, on the one hand, is a much more thermally stable substance as compared to HLT_3_ × MeNH_2_ and HLT_3_ ×BuNH_2_. On the other hand, the decomposition of its organic part starts at approximately 250 °C, which is lower than the temperature of the HLT_3_ × MeOH decomposition. This inexplicitly indicates a possible noncovalent nature of the obtained HLT_3_ × MEA compound.

The resulting compositions assigned to the obtained hybrids, as well as the temperature of their decomposition, as determined from the thermal analysis, are listed in Table 2.

### 2.4. Scanning Electron Microscopy

**Figure 4:**
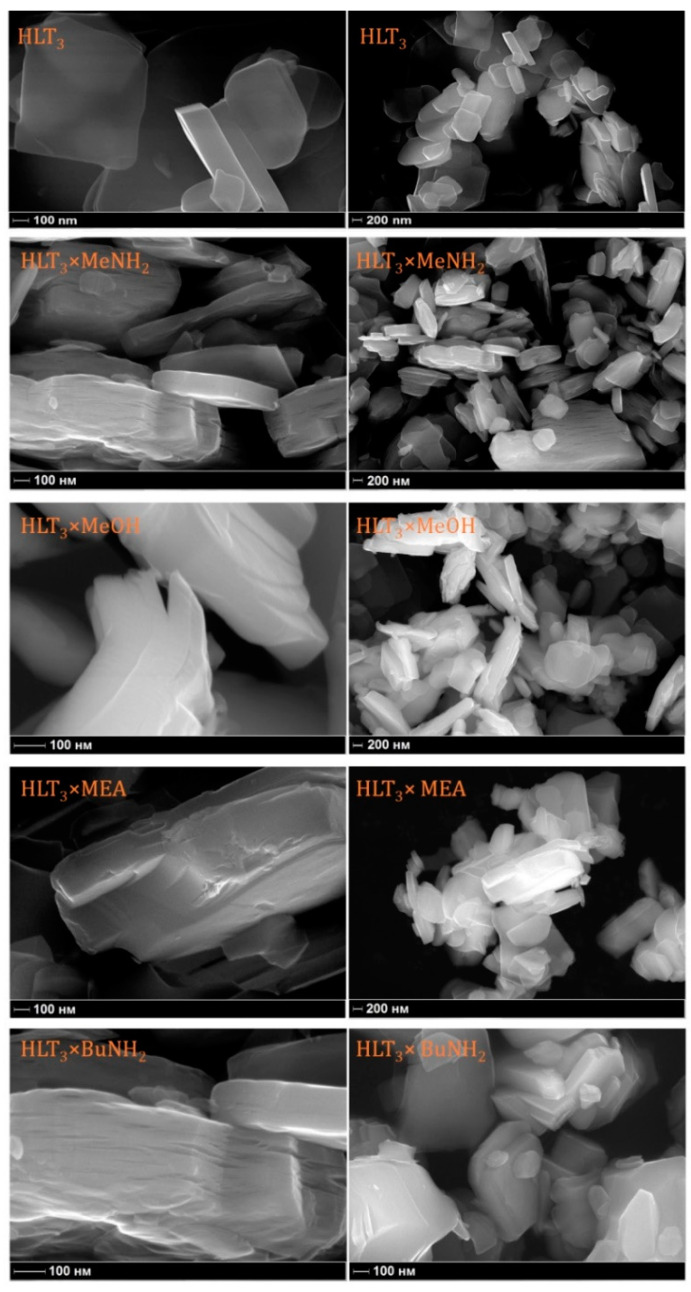
SEM images of the initial HLT_3_ and organic-inorganic HLT_3_ × MeOH, HLT_3_ × MeNH_2_, HLT_3_ × MEA, and HLT_3_ × BuNH_2_ compounds.

According to the scanning electron microscopy (SEM) data (Figure 4), during the intercalation and grafting reactions, both sizes and the lamellar forms of the particles are predominantly retained. Although the SEM images of the HLT_3_ × MeNH_2_ and HLT_3_ × BuNH_2_ organic-inorganic derivatives clearly confirm their incipient lamination, no other significant morphological changes are observed. Thus, all the reactions of the hybrid formation follow the topochemical mechanism: the structure of the inorganic part of the hybrids stays almost native and, consequently, saves all the useful properties of the initial perovskite-like compound, which may be combined with those of an organic part, giving new materials.

### 2.5. NMR Studies

To establish whether the hybrids are intercalation compounds or grafted derivatives, ^1^H-, ^13^C-, and ^15^N-NMR studies were performed. ^1^H-NMR has been used to characterize the distribution of mobile protons between different fractions in confined geometry. Different hydrogen bond environments exhibit different chemical shifts when the proton exchange between the fractions is slow within a time scale of ms [57]. ^1^H-NMR chemical shift of a mobile proton can be as large as 21.7 ppm [58]. ^1^H-NMR chemical shift of bulk water is 4.8 ppm. The chemical shift of an average H^+^/H_2_O signal depends on the concentration of H^+^ and, in a binary mixture, is larger than 5 ppm. Isolated surface hydroxyl groups resonate below 2 ppm [59]. The chemical shift of an average HO/H_2_O signal depends on the amount of water and, in a binary mixture, is smaller than 5 ppm. ^13^C-NMR has been used to characterize the rotational mobility of organic moieties. Chemical shift is a tensor quantity. The anisotropy of this tensor decreases if a moiety reorients fast within a time scale of ms [60]. ^15^N-NMR has been used to characterize the protonation state of amino moieties [61].

#### 2.5.1. NMR Studies of the HLT_3_ × MeOH Derivative

**Figure 5:**
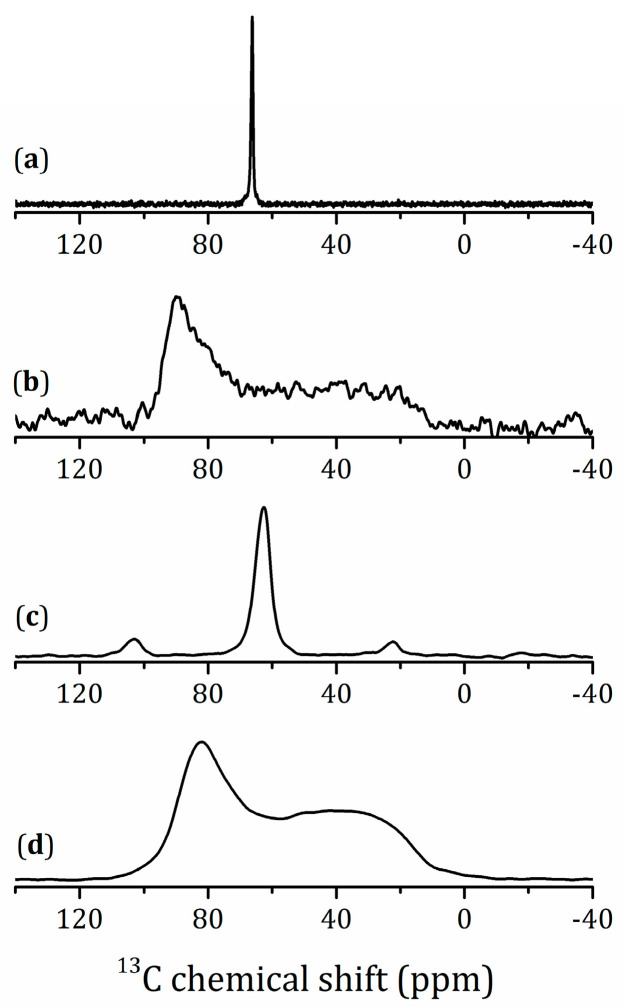
^13^C{^1^H} cross-polarization (CP) NMR spectra of HTL_3_ × MeOH under magic angle spinning (MAS) at ν_rot_ = 14 kHz (**a**) and static (**b**) conditions in magnetic field 9.4 T and ^13^C-NMR spectra of Ti(CH_3_O)_4_ under MAS at ν_rot_ = 5 kHz (**c**) and static (**d**) conditions in magnetic field 7 T.

Figure 5a,b represents ^13^C{^1^H} cross-polarization (CP) NMR spectra of the methanol derivative recorded under magic angle spinning (MAS) and static conditions, respectively. The ^13^C{^1^H} CP/MAS spectrum shows a line at 66.2 ppm. The ^13^C chemical shift tensor of this O-CH_3_ moiety is axially symmetric; its span is about 100 ppm. Therefore, this moiety cannot change its spatial orientation within the time scale of ms. The value of its isotropic chemical shift is low-field-shifted as compared to that of methanol (49 ppm). In contrast, the isotropic chemical shift, the symmetry, and the span of the tensor under discussion are similar to that of the O-CH_3_ moiety of Ti(CH_3_O)_4_ (Figure 5c,d). Therefore, the O-CH_3_ moieties of the methanol derivative are covalently bonded to the inorganic matrix, Ti-O-CH_3_.

**Figure 6:**
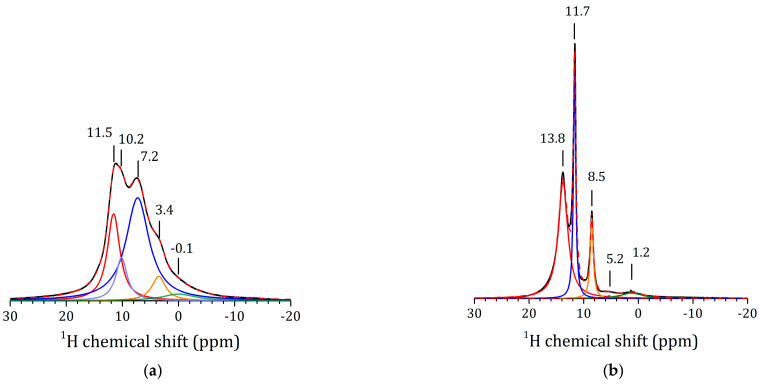
^1^H MAS NMR spectra of HLT_3_ × MeOH (**a**) and HLT_3_ (**b**) and their decomposition. Dashed lines show the total fit.

Figure 6a shows the ^1^H MAS NMR spectrum of the methanol derivative. The spectral lines are very broad, even at 14 kHz of spinning frequency. Consequently, both the rotational diffusion of the CH_3_ moieties and proton exchange are slow in this sample. The spectrum can be deconvoluted into three main peaks. The line at 3.4 ppm can be attributed to the CH_3_ moieties. The lines at 11 and 7 ppm can be attributed to the interlayer H^+^ in regular sites in water-poor and water-rich environments, respectively [50,62]. These results can be compared to the ^1^H MAS NMR spectrum of H_2_La_2_Ti_3_O_10_(H_2_O)_0.07_ (Figure 6b). In this sample, the lines corresponding to the water-poor (11.7 ppm) and water-rich (8.5 ppm) environments are narrow, which means that proton mobility in this environment is high. The line at 13.8 ppm can be attributed to the interlayer H^+^ in a water-free site. The mobility of these species is restricted, and the line is broad. Note that the modification of the inorganic matrix strongly affects the mobility and the distribution of the interlayer H^+^. The high-field signals in these spectra can belong to residual TiOH groups and surface-bound water [63,64,65].

#### 2.5.2. NMR Studies of the HLT_3_ × MeNH_2_ and HLT_3_ × BuNH_2_ Derivatives

**Figure 7:**
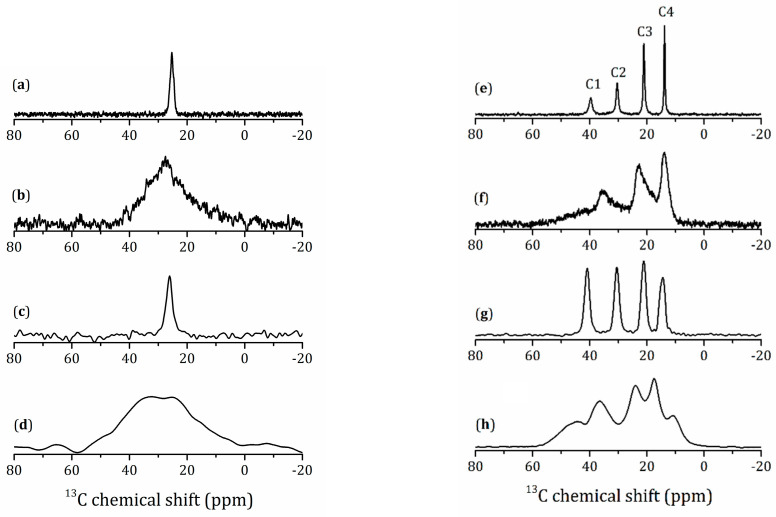
^13^C{^1^H} CP NMR spectra of HLT_3_ × MeNH_2_ under MAS at ν_rot_ = 14 kHz (**a**) and static (**b**) conditions in magnetic field 9.4 T. ^13^C-NMR spectra of MeNH_2_HCl under MAS at ν_rot_ = 5 kHz (**c**) and static (**d**) conditions in magnetic field 7 T. ^13^C{^1^H} CP NMR spectra of HLT_3_ × BuNH_2_ under MAS at ν_rot_ = 14 kHz (**e**) and static (**f**) conditions in magnetic field 9.4 T. ^13^C-NMR spectra of BuNH_2_HCl under MAS at ν_rot_ = 5 kHz (**g**) and static (**h**) conditions in magnetic field 7 T.

^13^C-NMR spectra of the methylamine and *n*-butylamine derivatives are shown in Figure 7. For HLT_3_ × MeNH_2_ in the ^13^C{^1^H} CP/MAS spectrum, only one line at 25.3 ppm is observed (see Figure 7a), which is in fair agreement with the position of the methyl carbon for the methylammonium cation.

The static spectrum, Figure 7b, represents the characteristic powder pattern on an anisotropic magnetic shielding tensor with a nonaxial symmetry. Therefore, the reorientational diffusion of these methylamine molecules is slow on the NMR time scale of ms. This gives evidence for the intercalation of methylamine. Note that this peak represents averaging over a variety of slightly different tensors, because the local environment of each individual methylammonium cation is different. Therefore, the principal values of these magnetic shielding tensors cannot be evaluated from this spectrum. This situation is typical for amorphous solids [66], surface functional groups [67], and molecules in confined geometry [68].

The spectra for powder CH_3_NH_2_HCl are given for a comparison in Figure 7c,d. The line position is almost unchanged. However, the powder pattern shows different anisotropy that can be related to the amorphous nature of this CH_3_NH_2_HCl sample. We are not aware about any data on the existence of crystalline CH_3_NH_2_HCl.

The ^13^C{^1^H} CP/MAS NMR spectrum of the *n*-butylamine derivative exhibits four lines at 39.7, 30.4, 21.1, and 13.8 ppm (Figure 7e). This figure shows the attribution of the lines to the four chemically different carbons of *n*-butylamine. The numeration starts from the amino group. It is obvious that the line width of these peaks decreases with the distance from the amino group. This trend remains valid for the anisotropy of the corresponding tensors (Figure 7f). In contrast, both the line width of the isotropic peaks and the anisotropy of the tensors are similar for all four carbons in a polycrystalline BuNH_2_HCl (Figure 7g,h). Therefore, the reorientational mobility of the *n*-butyl moieties in HLT_3_ × BuNH_2_ is not sterically hindered.

The ^15^N{^1^H} CP/MAS NMR spectra of HLT_3_ × MeNH_2_ and HLT_3_ × BuNH_2_ show single lines at 32.52 ppm and 44.01 ppm, respectively. The ^15^N chemical shift δ(^15^N) of neat MeNH_2_ and (MeNH_3_)^+^ in water is ~3 and 35 ppm, respectively [69,70]. The δ(^15^N) of neat BuNH_2_ and (BuNH_3_)^+^Cl^−^ in methanol is ~22 and 34 ppm, respectively [71,72].

Altogether, it points out that, in the both methylamine and *n*-butylamine derivatives, the organic species are intercalated in the form of methyl- or butylammonium cations. Their (–NH_3_)^+^ moieties are immobilized due to electrostatic interactions with negatively charged perovskite layers, while the aliphatic residues remain flexible.

**Figure 8:**
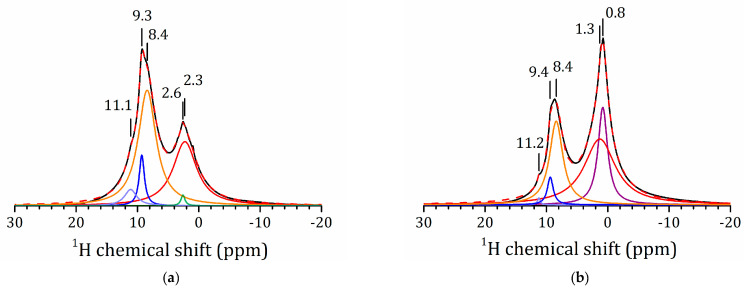
^1^H MAS NMR spectra of HLT_3_ × MeNH_2_ (**a**) and HLT_3_ (**b**) and their decomposition. Dashed lines show the total fit.

The ^1^H MAS NMR spectra of these amine derivatives are shown in Figure 8. The lines of the aliphatic residues are broad and overlap. Consequently, the libration of these residues is sterically hindered. The chemical shifts of the interlayer H^+^ and the –(NH_3_)^+^ moieties are in the range 11–8 ppm. The presence of several spectral lines indicates that the distribution of the organic species in the samples is not homogeneous.

#### 2.5.3. NMR Studies the HLT_3_ × MEA Derivative

**Figure 9:**
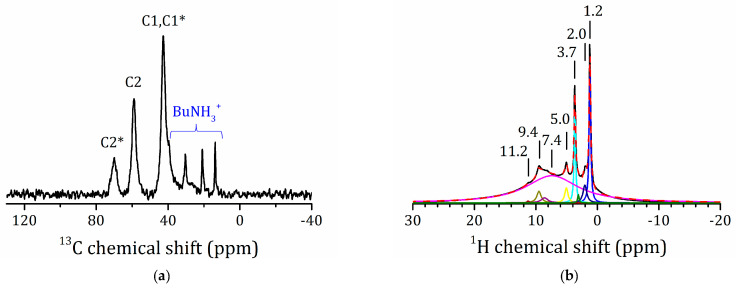
^13^C{^1^H} CP/MAS (**a**) and ^1^H MAS NMR (**b**) spectra of HLT_3_ × MEA.

The decomposition temperature of the monoethanolamine derivative is similar to that of the methanol derivative and is much higher as compared to that of the methylamine and *n*-butylamine derivatives (Table 2). This suggests that the methylene carbons of the monoethanolamine residues are covalently bound to the lattice oxygens. Figure 9a shows the ^13^C{^1^H} CP/MAS NMR spectrum of HLT_3_ × MEA.

Besides the peaks of the residual BuNH_3_^+^, there are three new peaks at 42.6, 58.9, and 69.9 ppm. The first of these peaks obviously belongs to the –CH_2_-NH_3_^+^ carbon atom (C1 carbon) [73]. The corresponding peak of BuNH_3_^+^ has a similar chemical shift. The two other lines should be attributed to the –O-CH_2_- carbon atom. Therefore, in this sample, two different substituents at the oxygen atom are possible. The ^13^C chemical shifts of isopropanol ((CH_3_)_2_CHOH) and titanium isopropoxide (Ti(OCH(CH_3_)_2_)_4_) are 25.3/63.7 and 26.6/76.2 ppm, respectively [74]. Consequently, the conversion of a HOC- moiety into a –TiOC- one has a small effect on the chemical shift of remote carbon nuclei. The signal of the –OC- moiety will be shifted to lower field by about 10 ppm. We conclude that the peak at 58.9 ppm in the spectrum of the monoethanolamine derivative belongs to the C2 carbon of intercalated protonated MEA, HOCH_2_CH_2_NH_3_^+^ [73]. In contrast, the peak at 69.9 ppm belongs to TiOCH_2_CH_2_NH_3_^+^ residues covalently bound to the inorganic matrix; we denote it as C2*. Obviously, the peak of C1* of the grafted TiOCH_2_CH_2_NH_3_^+^ coincides with that of C1 of the intercalate.

The ^1^H spectrum of HLT_3_ × MEA is not very informative (Figure 9b). The broad line centered at 7.4 ppm includes all types of mobile protons in different environments and is very broadened, and it is impossible to select contributions from the interlayer H^+^, the –(NH_3_)^+^, and –NH_2_ moieties. The narrow lines at 3.7 and 1.2 ppm are more likely from mobile moieties of intercalated molecules.

#### 2.5.4. VCT Experiment to Study the Dynamics of Organic Molecules

**Figure 10:**
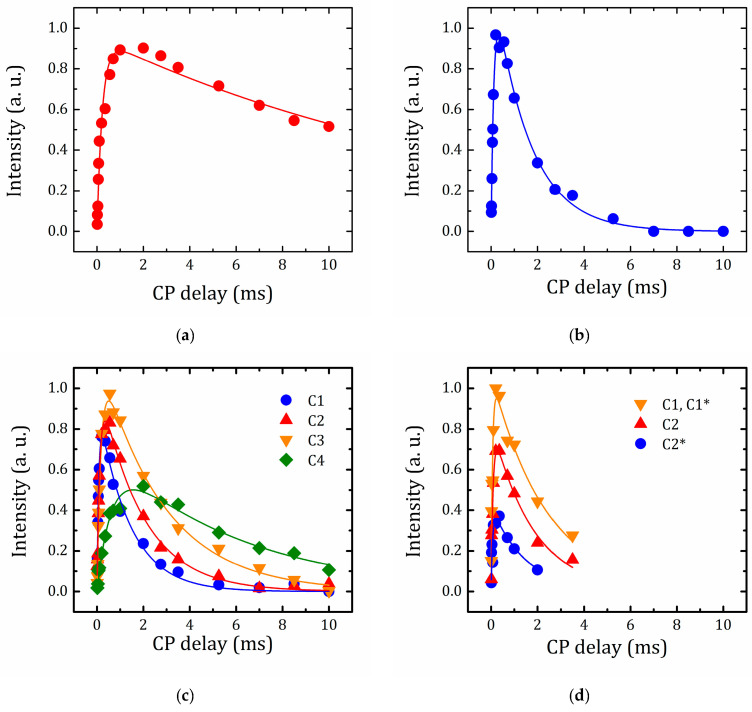
Integral peak intensity for carbon atoms in HLT_3_ × MeOH (**a**), HLT_3_ × MeNH_2_ (**b**), HLT_3_ × BuNH_2_ (**c**), and HLT_3_ × MEA (**d**).

To study the dynamics of the organic molecules within the interlayer space, a ^13^C{^1^H} CP/MAS experiment with variable contact time (VCT) was carried out. Figure 10 represents the intensity of the carbon peaks of the organic molecules introduced in HLT_3_ as a function of contact time ^1^H-^13^C. The dependence of the signal intensity on the contact period during VCT can be described by the following Equation (1) [75]:

(1) $$I\;=\;I_{0}{\left(1\;-\;\frac{T_{\mathrm{CH}}}{T_{1\rho }\left(H\right)}\right)}^{-1}\cdot \left[\exp\left(-\frac{\tau_{\mathrm{cp}}}{T_{1\rho }\left(H\right)}\right)-\exp\left(-\frac{\tau_{\mathrm{cp}}}{T_{\mathrm{CH}}}\right)\right]$$

where *T*_CH_ is the cross-polarization time of a chemical group generating the corresponding NMR signal. It determines the growing part of the signal intensity and reflects the efficiency of the cross-polarization transfer from the ^1^H to ^13^C nuclei. This parameter is normally different for carbons belonging to different functional groups. Its value, on the one hand, is determined by the number of protons near the carbon nucleus and, on the other hand, by the rigidity of the carbon bonding with the “lattice”. The signal decay is governed by *T*_1ρ_(H), which is the proton longitudinal relaxation time (in the rotating frame) associated to the corresponding functional group.

The *T*_CH_ and *T*_1ρ_(H) parameters for all the carbon sites of the studied organic molecules, as determined from the dependencies shown in Figure 10 applying Equation (1), are listed in Table 3.

For the carbons of BuNH_3_^+^, as, when moving away from the nitrogen atom, both *T*_CH_ and *T*_1ρ_(H) values grow up reflecting the increasing mobility. This is in-line with gradually decreasing the ^13^C MAS NMR line width from 0.64 ppm for C1 to 0.20 ppm for C4 (Figure 7e). The carbon atom of the methyl group in the methanol derivative, which is covalently bounded to the lattice oxygen, exhibits the highest *T*_1ρ_(H) value and the intermediate *T*_CH_ one, as compared to the methyl groups very mobile in BuNH_3_^+^ and rather slow in MeNH_3_^+^_._ It means that the rotational motion of the CH_3_ group is relatively fast.

For the HLT_3_ × MEA derivative, in which both intercalated cations and grated species coexist, *T*_CH_ and *T*_1ρ_(H) values for both types of carbons are close, within the experiment error, to those for C1 of BuNH_3_^+^.

## 3. Materials and Methods

The layered perovskite H_2_La_2_Ti_3_O_10_·*x*H_2_O (HLT_3_), where *x* is an amount of intercalated water, was synthesized using K_2_La_2_Ti_3_O_10_ as a precursor. K_2_La_2_Ti_3_O_10_ was prepared by solid-phase synthesis, as described in Reference [50]. The protonation of K_2_Ln_2_Ti_3_O_10_ was carried out by the three-step procedure. Initially, the oxide was kept in moist air for 12 h. Then, it was treated with an excess of water (200 mL per 1 g of the oxide) for 1 h and, hereafter, with an excess of 0.1-M HNO_3_ (200 mL per 1 g of the oxide) for 12 h. After centrifugation, the product was dried under ambient pressure. The tetragonal lattice parameters of the protonated form obtained were found to be *a* = 3.809 and *c* = 27.55 Å, which is consistent with the values in earlier reports [4,76]. The elimination of K^+^ was confirmed by thermogravimetric analysis (TG) and, additionally, by energy-dispersive X-ray (EDX), which showed the lack of a potassium line. Calculations performed by the reported technique [7,77] showed that no less than 95% of the alkali cations were replaced by protons.

Single-phase inorganic-organic hybrids were synthesized under optimal conditions found in the preliminary experiments (see Supplementary Materials). The methylamine derivative, H_2_La_2_Ti_3_O_10_ × MeNH_2_ (HLT_3_ × MeNH_2_), was obtained by the reacting of H_2_La_2_Ti_3_O_10_ with an excess of 34% aqueous methylamine solution at 60 °C for 7 days under continuous stirring. Taking into account that large amine or alcohol molecules are difficult to introduce into H_2_La_2_Ti_3_O_10_, the hybrid structures containing *n*-butylamine and methanol were synthesized using the methylamine derivative as a precursor. To synthesize the *n*-butylamine derivative, H_2_La_2_Ti_3_O_10_ × BuNH_2_ (HLT_3_ × BuNH_2_), a mixture of the HLT_3_ × MeNH_2_ powder and an excess of 90% *n*-butylamine aqueous solution was stirred at room temperature for 7 days. The adduct with methanol, H_2_La_2_Ti_3_O_10_ × MeOH (HLT_3_ × MeOH), was obtained by a solvothermal reaction between the HLT_3_ × BuNH_2_ derivative and methanol at 100 °C for 10 days. The products were separated via centrifugation, washed with acetone, and dried in a desiccator. The monoethanolamine hybrid, H_2_La_2_Ti_3_O_10_ × MEA (HLT_3_ × MEA), was prepared based on the *n*-butylamine derivative HLT_3_ × BuNH_2_ at 25 °C in 1 d using 90% monoethanolamine in water.

XRD patterns were obtained on the Rigaku Miniflex II diffractometer (Tokyo, Japan) (CuK_α_ radiation, angle range 2θ = 3–60°, scanning rate 10°/min, step 0.01°). The lattice parameters were calculated based on all the reflections observed using DiffracPlus Topas software (version 4.2). Raman spectra were obtained on the Bruker Senterra spectrometer (Karlsruhe, Germany) (spectral range 100–4000 cm^−1^, incident laser 488 nm 20 mW, and spectrum accumulation time 10 s). Fourier-transform infrared (IR) absorption spectra were recorded on the Shimadzu IRAffinity-1 spectrometer (Kyoto, Japan) (spectral range 400–4000 cm^−1^, tableting in KBr). The amounts of carbon, hydrogen, and nitrogen in the hybrids were determined by the elemental C,H,N analysis on the Euro EA3028-HT analyzer (Redavalle, Italy). Simultaneous thermal analysis coupled with the mass spectrometric detection of gases evolved (STA-MS) was carried out on the Netzsch STA 409 CD-QMS 403/5 Skimmer system (Netzsch-Gruppe, Selb, Germany) using an air-containing (oxidative) atmosphere. Calculation of quantitative compositions of the inorganic-organic hybrids from STA-MS data was based on the direct proportionality of ion currents to the quantities of gases evolved and matching integrated ion currents with corresponding mass losses. The final compositions were established using results of the C,H,N analysis and STA-MS in total. The morphology of the samples was investigated by SEM on the Zeiss Merlin scanning electron microscope.

The solid-state ^1^H-, ^13^C-, and ^15^N-NMR spectra were recorded on the Bruker Avance III 400WB spectrometer (Bruker Corporation, Billerica, MA, USA) using the MAS technique with a double-resonance 4-mm probe. ^1^H-NMR spectra were recorded at ν_rot_ = 14 kHz and the relaxation delay of 120 s. To increase the intensity of ^13^C and ^15^N signals, a cross-polarization (CP) was applied. Static ^13^C{^1^H} CP and ^13^C{^1^H} CP/MAS spectra (ν_rot_ = 14 kHz) were recorded at a relaxation delay of 5 s and a contact period τ_cp_ = 2 ms (for HLT_3_ × MEA, τ_cp_ = 0.7 ms was used). ^13^C{^1^H} CP/MAS variable contact time (VCT) experiments were performed with τ_cp_ varied between 10 and 10,000 µs. ^15^N{^1^H} CP/MAS spectra were obtained at ν_rot_ = 12.5 kHz. Tetramethylsilane was used as an internal standard for ^1^H and ^13^C, and ammonium chloride was used as an external standard for ^15^N (δ(^15^NH_4_Cl_cryst_) = 39.3 ppm relative to the liquid ammonia and can be converted into the nitromethane scale using the relations δ(CH_3_^15^NO_2_) = δ(^15^NH_4_Cl) − 338.1 ppm [61]. ^13^C NMR spectra of crystalline MeNH_2_HCl, BuNH_2_HCl, and Ti(CH_3_O)_4_ were recorded at 7 T on the Infinityplus spectrometer system (Agilent, Santa Clara, CA, USA) using a Chemagnetics-Varian 6-mm CP/MAS probe. These ^13^C{^1^H} CP/MAS data were acquired using a relaxation delay of 5 s and ν_rot_ = 5 kHz.

## 4. Conclusions

This study shows that the interlayer spaces of protonated perovskite-like titanates H_2_Ln_2_Ti_3_O_10_ (Ln = La and Nd) can be modified with the simplest representatives of amines (methylamine and *n*-butylamine), alcohols (methanol), and amino alcohols (monoethanolamine), giving inorganic-organic hybrids. The synthetic pathways of these hybrids were studied in a wide range of conditions using both standard laboratory techniques and solvothermal/solvothermal microwave methods. The optimal pathways of their preparation are summarized. Specifically, the methylamine hybrid, which was obtained by a one-step direct reaction, is a convenient precursor for the preparation of other types of hybrids. Thus, pure *n*-butylamine, methanolic, and monoethanolamine hybrids can be easily obtained using the methylamine hybrid as the precursor.

The ^1^H, ^13^C, and ^15^N solid-state NMR spectroscopy and ^13^C{^1^H} VCT experiment, applied to study the bonding type of the obtained organic-inorganic hybrids and mobility of the organic components, unambiguously indicate that, in the methanolic hybrid, the organic residues are covalently bound to the inorganic matrix. In contrast, in the methylamine and *n*-butylamine hybrids, the organic molecules are intercalated into the inorganic matrix in a cationic form. The interlayer space of the monoethanolamine hybrid contains both the covalently bound and intercalated organic species.

These conclusions are supported by the vibrational spectroscopy data and thermal analysis. The thermal stability of the studied hybrids increases in a sequence: methylamine/*n*-butylamine → monoethanolamine → methanol. The methanolic and monoethanolamine hybrids, due to their high thermal stability, can apparently be obtained in a hydrous form by thermal dehydration at 125–150 °C.

## Figures and Tables

**Table 1:** Lattice parameters (*a* and *c* indexed in the tetragonal system) and interlayer distances *d* of the initial protonated titanate and its inorganic derivatives. HLT_3_: H_2_La_2_Ti_3_O_10_·*x*H_2_O.

Sample	*a* (Å)	*c* (Å)	*d* (Å)
HLT_3_	3.809	27.55	13.78
HLT_3_ × MeNH_2_	3.830	39.94	18.97
HLT_3_ × BuNH_2_	3.828	24.23	24.23
HLT_3_ × MeOH	3.800	35.40	17.70
HLT_3_ × MEA	3.832	38.97	19.49

**Table 2:** The composition and decomposition temperature (*T*_d_) for the studied compounds. TG: thermal gravimetric.

Sample	Composition	Total Mass Loss in TG (%)	*T*_d_ (°C)
HLT_3_	H_2_La_2_Ti_3_O_10_(H_2_O)_0.05_	3.01	280
HLT_3_ × MeNH_2_	H_2_La_2_Ti_3_O_10_(MeNH_2_)_0.75_(H_2_O)_0.20_	7.29	75
HLT_3_ × BuNH_2_	H_2_La_2_Ti_3_O_10_(BuNH_2_)_0.60_(H_2_O)_0.20_	10.3	75
HLT_3_ × MeOH	H_1.3_La_2_Ti_3_O_9.3_(MeO)_0.75_(H_2_O)_0.05_	4.75	300
HLT_3_ × MEA	H_2_La_2_Ti_3_O_10_(MEA)_0.65_(H_2_O)_0.10_	9.47	250

**Table 3:** Values of *T*_CH_ and *T*_1ρ_(H) for the specific ^13^C nuclei with an isotropic chemical shift δ_iso_ in the studied hybrids, as derived from ^1^H-^13^C cross-polarization/magic angle spinning (CP/MAS) NMR measurements.

Compound	Carbon Site	δ_iso_ (ppm)	*T*_CH_ (μs)	*T*_1ρ_(H) (ms)
HLT_3_ × MeOH	C	66.2 ± 0.1	233 ± 29	17.1 ± 2.8
HLT_3_ × MeNH_2_	C	25.2 ± 0.1	131 ± 10	1.5 ± 0.1
HLT_3_ × BuNH_2_	C1	39.7 ± 0.1	79 ± 6	1.3 ± 0.1
	C2	30.4 ± 0.1	129 ± 9	1.8 ± 0.1
	C3	21.1 ± 0.1	182 ± 13	2.3 ± 0.2
	C4	13.8 ± 0.1	700 ± 93	5.8 ± 0.6
HLT_3_ × MEA	C1,C1*	42.4 ± 0.1	64 ± 9	2.3 ± 0.4
	C2	58.7 ± 0.1	87 ± 11	1.8 ± 0.3
	C2*	69.4 ± 0.1	67 ± 14	1.5 ± 0.6

## Supplementary Materials

The following are available online: Figure S1: XRD patterns of (a) HLT_3_ and products of low-temperature reactions between HLT_3_ and methylamine under various conditions: (b) 1 d at 25 °C, (c) 1 d at 60 °C, (d) 7 d at 25 °C, (e) 7 d at 60 °C, (f) 14 d at 25 °C, and (g) 14 d at 60 °C. Figure S2: XRD patterns of (a) HLT_3_ and products of solvothermal and solvothermal microwave reactions between HLT_3_ and methylamine of various durations at 100 °C: (b) ST 1 d, (c) ST 7 d, (d) STMW 1 h, (e) STMW 1 d, and (f) STMW 3 d. Figure S3: XRD patterns of (a) HLT_3_ and products of low-temperature reactions between HLT_3_ and *n*-butylamine under various conditions: (b) 1 d at 25 °C, (c) 1 d at 60 °C, (d) 7 d at 25 °C, and (e) 7 d at 60 °C. Figure S4: XRD patterns of (a) HLT_3_ and products of solvothermal (ST) and solvothermal microwave (STMW) reactions between HLT_3_ and *n*-butylamine under various conditions: (b) ST 7 d 100 °C, (c) STMW 1 h 100 °C, (d) STMW 1 h 150 °C, and (e) STMW 1 d 100 °C. Figure S5: XRD patterns of (a) HLT_3_ × MeNH_2_ and products of reactions between HLT_3_ × MeNH_2_ and methanol: (b) 7 d at 60 °C, (c) 5 d under solvothermal conditions at 100 °C, and (d) 1 d under solvothermal microwave conditions at 100 °C. Figure S6: IR spectra of (a) HLT_3_, (b) HLT_3_ × MeNH_2_, (c) HLT_3_ × BuNH_2_, (d) HLT_3_ × MeOH, and (e) HLT_3_ × MEA. Figure S7: STA-MS data for methylamine hybrid HLT_3_ × MeNH_2_. Figure S8: STA-MS data for *n*-butylamine hybrid HLT_3_ × BuNH_2_. Figure S9: STA-MS data for methanolic hybrid HLT_3_ × MeOH. Figure S10: STA-MS data for monoethanolamine hybrid HLT_3_ × MEA. Table S1: Conditions of experiments on the optimization of the hybrid synthesis.
