# Supplementary material for: NMR Study of Intercalates and Grafted Organic Derivatives of H2La2Ti3O10
Source: Molecules. 2020 Nov 10;25(22):5229. doi: 10.3390/molecules25225229 (PMC7696603; doi:10.3390/molecules25225229)
Supplement: Supplementary file 1 [file molecules-25-05229-s001.pdf]

Supplementary Materials

for

NMR study of intercalates and grafted organic derivatives of $H_2La_2Ti_3O_{10}$

Marina G. Shelyapina [1,*], Oleg I. Silyukov[1], Irina P. Lushpinskaya[1], Sergey A. Kurnosenko[1], Anton S. Mazur[1], Ilya G. Shenderovich[2] and Irina A. Zvereva[1]

[1] Saint-Petersburg State University, 7/9 Universitetskaya nab., St. Petersburg 199034, Russia
[2] University of Regensburg, Universitätsstr. 31, 93040 Regensburg, Germany
* Correspondence: marina.shelyapina@spbu.ru

1. Study of reactivity with organic compounds

Table S1 recaps conditions of experiments carried out in order to establish suitable ways for obtaining of single-phase organic-inorganic hybrids. Before experiments, weighed protonated forms were thoroughly ground in an agate mortar. Amine hybrids, which were used as precursors, were taken without preliminary grinding. Low-temperature syntheses were conducted in hermetically sealed glass tubes with stirring, solvothermal experiments – in laboratory autoclaves, solvothermal-microwave ones – using a Berghof Speedwave 4 system with PTFE vessels. All the products were centrifuged, washed with distilled acetone and dried under ambient pressure. Formulae of hybrids below are merely conventions and do not show true compositions and types of bonding.

Table S1. Conditions of experiments on optimization of the hybrid synthesis

Synthesis of methylamine hybrid $HLT_3 \times MeNH_2$			
Method	Low-temperature synthesis	Solvothermal synthesis	Solvothermal-microwave synthesis
Precursors	HLT_3 (0.2 g)	HLT_3 (0.2 g)	HLT_3 (0.2 g)
Reaction medium	38% methylamine in water (10 ml)	38% methylamine in water (30 ml)	38% methylamine in water (30 ml)
Temperatures, °C	25, 60	100	100, 150
Duration	1 h – 14 d	1, 3, 7 d	1 h – 3 d

Synthesis of *n*-butylamine hybrid $HLT_3 \times BuNH_2$			
Method	Low-temperature synthesis	Solvothermal synthesis	Solvothermal-microwave synthesis
Precursors	HLT_3, $HLT_3 \times MeNH_2$ (0.2 g)	HLT_3 (0.2 g)	HLT_3 (0.2 g)
Reaction medium	90% *n*-butylamine in water (10 ml)	90% *n*-butylamine in water (30 ml)	90% *n*-butylamine in water (30 ml)
Temperatures, °C	25, 60	100	100, 150
Duration	1 – 7 d	7 d	1 h – 3 d

Synthesis of methanolic hybrid $HLT_3 \times MeOH$			
Method	Low-temperature synthesis	Solvothermal synthesis	Solvothermal-microwave synthesis
Precursors	$HLT_3 \times MeNH_2$, $HLT_3 \times BuNH_2$ (0.2 g)	$HLT_3 \times MeNH_2$, $HLT_3 \times BuNH_2$ (0.2 g)	HLT_3, $HLT_3 \times MeNH_2$, $HLT_3 \times BuNH_2$ (0.2 g)
Reaction medium	90% methanol in water (10 ml)	90% methanol in water (30 ml)	90% methanol in water (30 ml)
Temperatures, °C	60	100	75 – 200
Duration	5, 7 d	5 d	1 – 3 d

Synthesis of monoethanolamine hybrid $HLT_3 \times MEA$			
Method	Low-temperature synthesis	Solvothermal synthesis	Solvothermal-microwave synthesis
Precursors	HLT_3, $HLT_3 \times MeNH_2$, $HLT_3 \times BuNH_2$ (0.2 g)	HLT_3, $HLT_3 \times MeNH_2$, $HLT_3 \times BuNH_2$ (0.2 g)	HLT_3 (0.2 g)
Reaction medium	90% monoethanolamine in water (10 ml)	90% monoethanolamine in water (30 ml)	90% monoethanolamine in water (30 ml)
Temperatures, °C	25, 60	100	150, 200
Duration	1 – 14 d	7 d	1 d

Methylamine hybrid. The fact of methylamine intercalation is indicated by appearance of new reflections at 5° in XRD patterns of the products corresponding to their interlayer distances and by a decrease in the intensity of reflections at 6.5° corresponding to interlayer distance of the initial protonated form HLT_3 (Figure S1). However, complete proceeding of the intercalation, that is producing single-phase hybrid without noticeable impurities of the initial protonated compound, can be reached at room temperature only after 10−14 d treatment, or in 7 d at 60°C (Figure S1). An increase in temperature from 25°C to 60°C does not result in formation of impurity phases. Synthesis of the hybrids under solvothermal conditions also requires the same duration (7 d) (Figure S2). Moreover, the solvothermal-microwave method, which is known to demonstrate high efficiency in a number of intercalation and grafting reactions, does not allow obtaining pure hybrid $HLT_3 \times MeNH_2$ in short time (durations 3 d at 100°C were tested) and, consequently, expediency of its using in this case is doubtful (Figure S2). Thus, the suitable way of obtaining single-phase hybrid $HLT_3 \times MeNH_2$ (60°C, 7 d) is established.

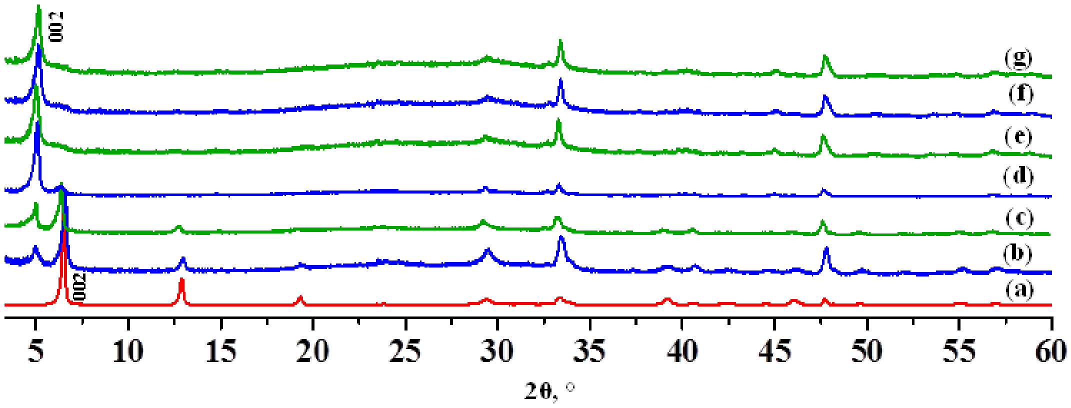

Figure S1. XRD patterns of (a) HLT_3, products of low-temperature reactions between HLT_3 and methylamine under various conditions (b) 1 d at 25°C, (c) 1 d at 60°C, (d) 7 d at 25°C, (e) 7 d at 60°C, (f) 14 d at 25°C, (g) 14 d at 60°C.

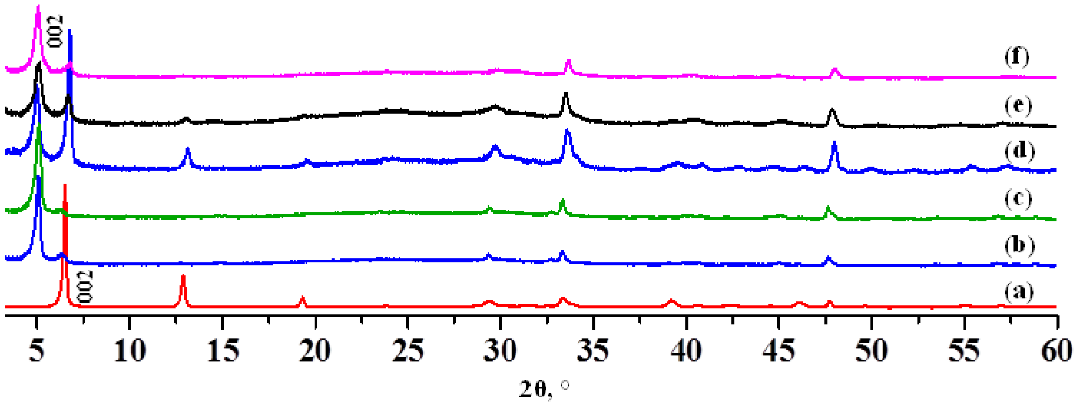

Figure S2. XRD patterns of (a) HLT_3, products of solvothermal and solvothermal-microwave reactions between HLT_3 and methylamine of various duration at 100°C (b) ST 1 d, (c) ST 7 d, (d) STMW 1 h, (e) STMW 1 d, (f) STMW 3 d

***n*-butylamine hybrid.** Direct low-temperature synthesis of pure *n*-butylamine hybrid $HLT_3 \times BuNH_2$ on the basis of the protonated forms, apparently, is not possible. Appearance of new reflections at 3.5° in XRD patterns of the products (Figure S3) reveals that *n*-butylamine intercalation does proceed but all the samples contain significant amounts of the initial protonated form. A rise in temperature to 60° does not lead to a noticeable increase in the yield of the hybrid. These difficulties in the direct preparation of the *n*-butylamine derivative are quite natural because of the larger *n*-butylamine size (6.8 Å) compared to methylamine (3 Å). Direct synthesis of the hybrid under solvothermal and solvothermal-microwave conditions also does not result in formation of the single-phase product (Figure S4). We revealed that pure hybrid $HLT_3 \times BuNH_2$ may be prepared on the basis of the methylamine derivative $HLT_3 \times MeNH_2$ at 25°C in 1 d. The increase in the duration of synthesis to 3 days does not lead to noticeable changes on the products XRD patterns.

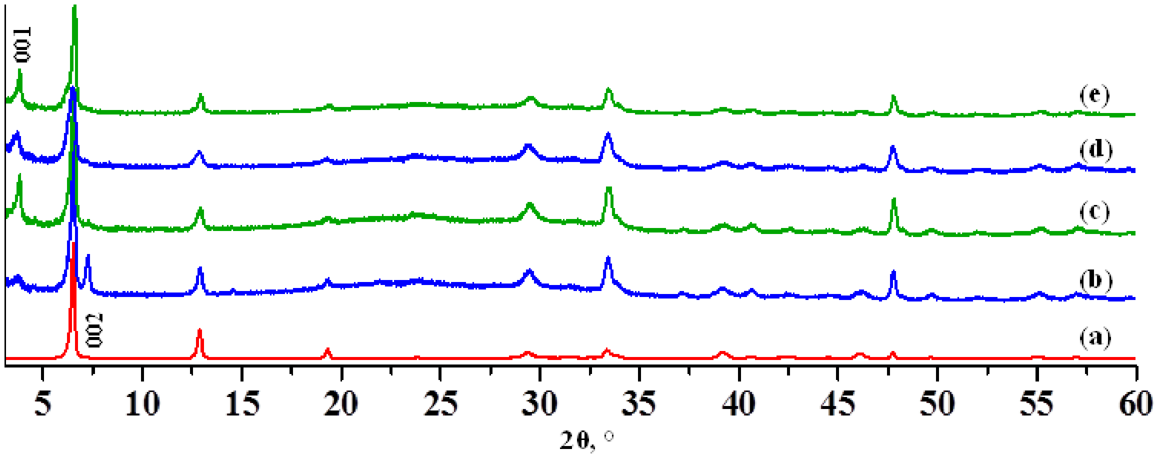

Figure S3. XRD patterns of (a) HLT_3, products of low-temperature reactions between HLT_3 and *n*-butylamine under various conditions (b) 1 d at 25°C, (c) 1 d at 60°C, (d) 7 d at 25°C, (e) 7 d at 60°C

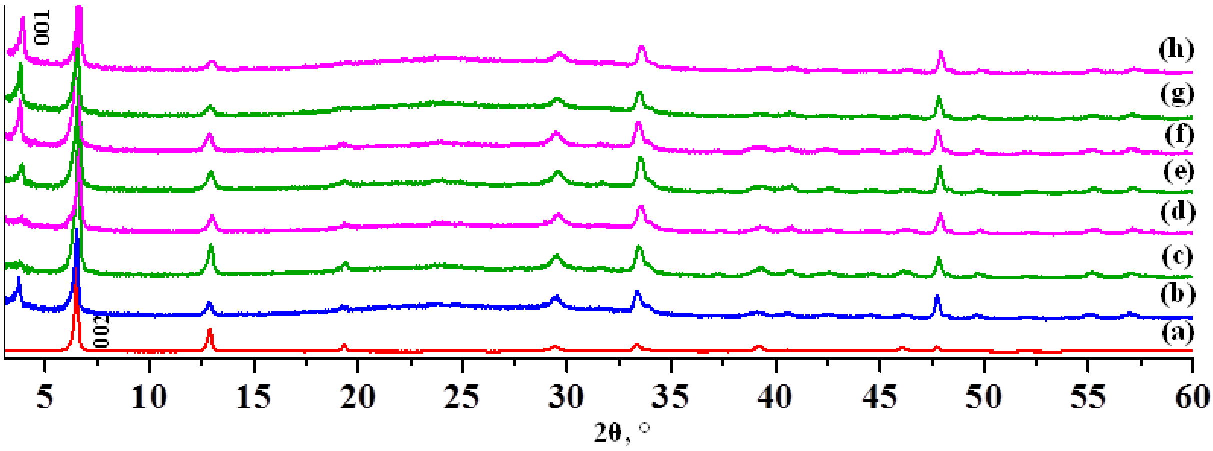

Figure S4. XRD patterns of (a) HLT_3, products of solvothermal (ST) and solvothermal-microwave (STMW) reactions between HLT_3 and *n*-butylamine under various conditions (b) ST 7 d 100°C, (c) STMW 1 h 100°C, (d) STMW 1 h 150°C, (e) STMW 1 d 100°C

Methanolic hybrid. Direct grafting of methanol into HLT_3 appears to impossible even under solvothermal and solvothermal-microwave conditions. XRD patterns of the samples obtained in this way (not shown) contain only reflections due to the initial protonated form indicating absence of the alcohol intercalation or grafting. Preparation of methanolic hybrid $HLT_3 \times MeOH$ is possible using amino derivatives $HLT_3 \times RNH_2$ (R = Me or Bu) as precursors. Solvothermal-microwave reactions lasting 1 d lead to formation of two-phase samples consisting of amino and methanolic derivatives that is indicated by bifurcation of reflections at 4.9–5.1° in XRD patterns of the products (Figure S5) and the presence of nitrogen in the samples detected by elemental C,H,N-analysis. Pure methanolic hybrid may be prepared at 60°C in 7 d in sealed tubes or at 100°C in 5 d in solvothermal autoclaves.

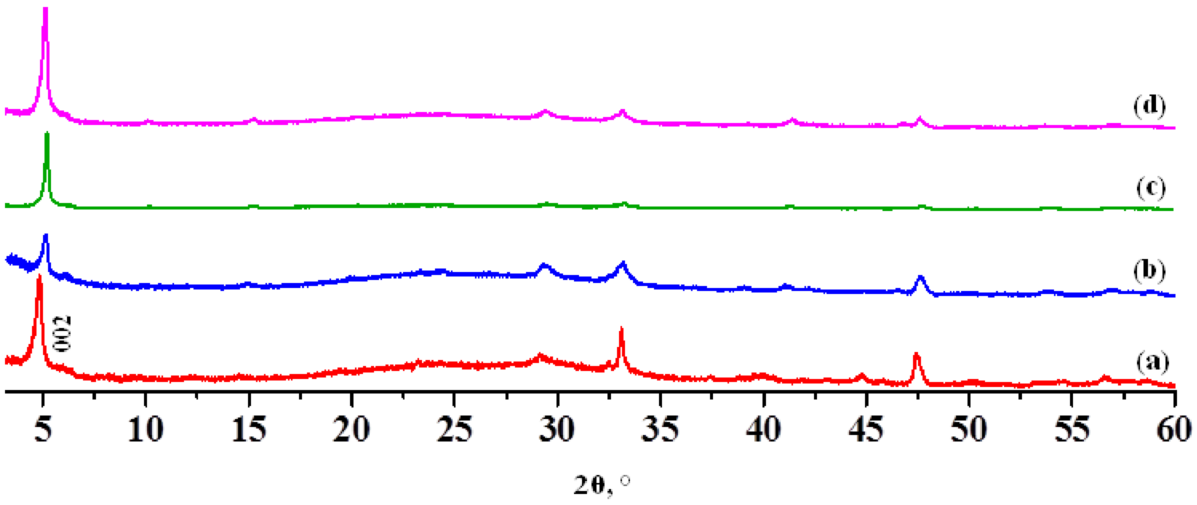

Figure S5. XRD patterns of (a) $HLT_3 \times MeNH_2$, products of reactions between $HLT_3 \times MeNH_2$ and methanol (b) 7 d at 60°C, (c) 5 d under solvothermal conditions at 100°C, (d) 1 d under solvothermal-microwave conditions at 100°C

Monoethanolamine hybrid. As in the case of the *n*-butylamine derivative, direct preparation of pure monoethanolamine hybrid $HLT_3 \times MEA$ using standard (60°C) or solvothermal-microwave methods (150, 200°C), apparently, is not possible: partial hybrid formation does proceed but yield of the target products is low. Variation of temperature and synthesis duration weakly affects the result. Pure hybrid $HLT_3 \times MEA$ may be prepared on the basis of amine derivatives $HLT_3 \times RNH_2$ (R = Me or Bu) at 25°C in 1 d. The increase in the duration of synthesis to 7 days does not affect structure and composition of the sample.

2. IR-absorption spectra of obtained compounds

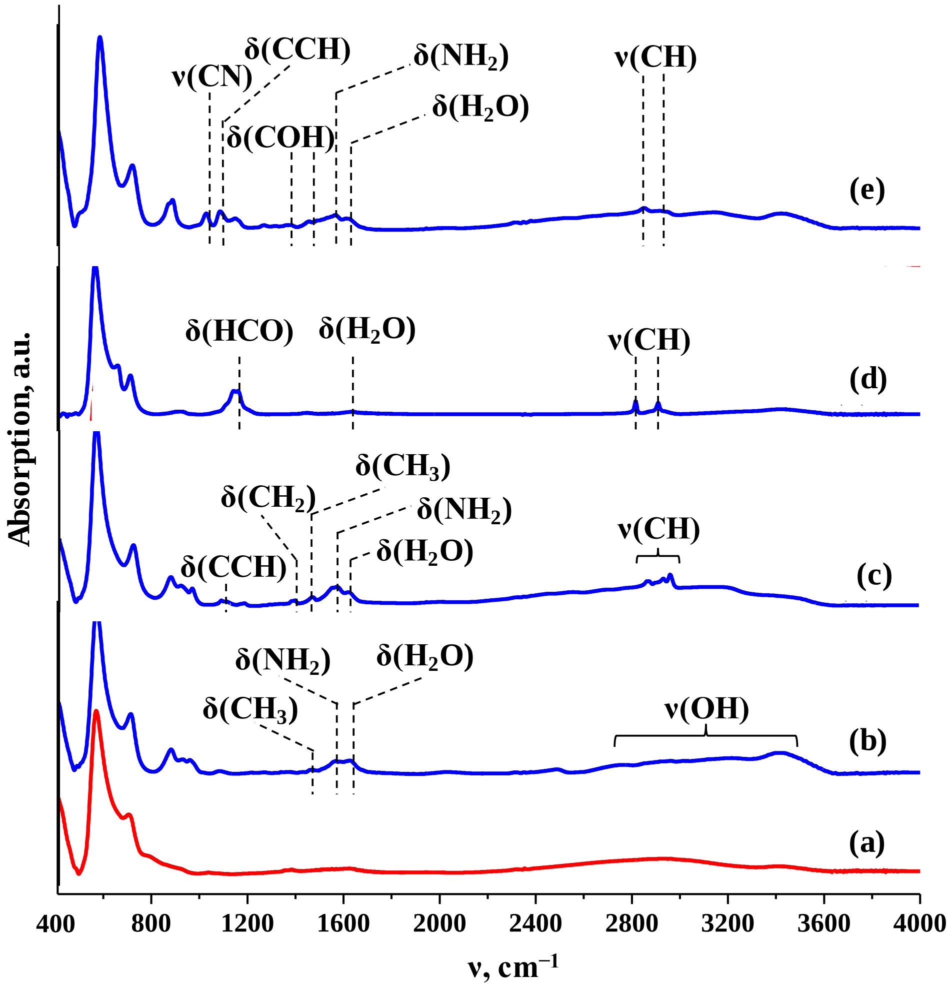

Figure S6. IR spectra of (a) HLT_3, (b) $HLT_3 \times MeNH_2$, (c) $HLT_3 \times BuNH_2$, (d) $HLT_3 \times MeOH$, (e) $HLT_3 \times MEA$

3. Simultaneous thermal analysis of obtained compounds coupled with masspectrometry indetification of evolved gases

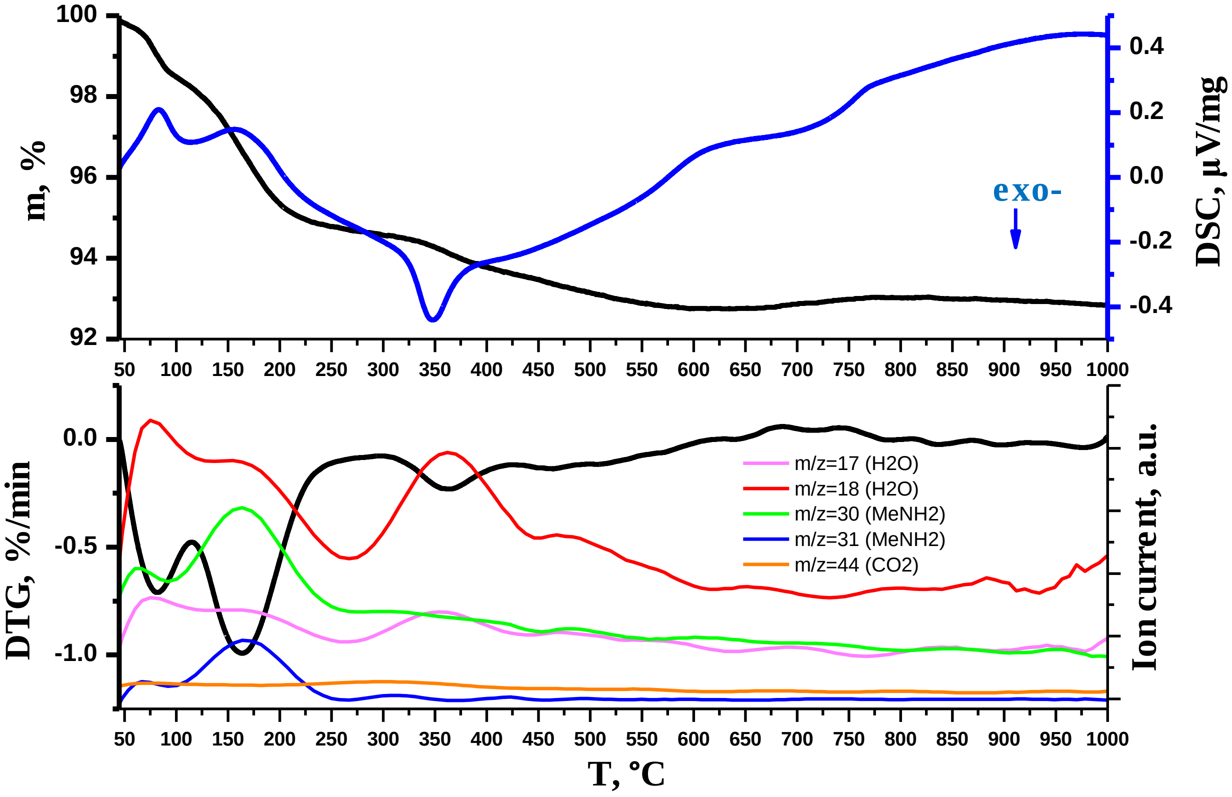

Figure S7. STA-MS data for methylamine hybrid $HLT_3{\times}MeNH_2$

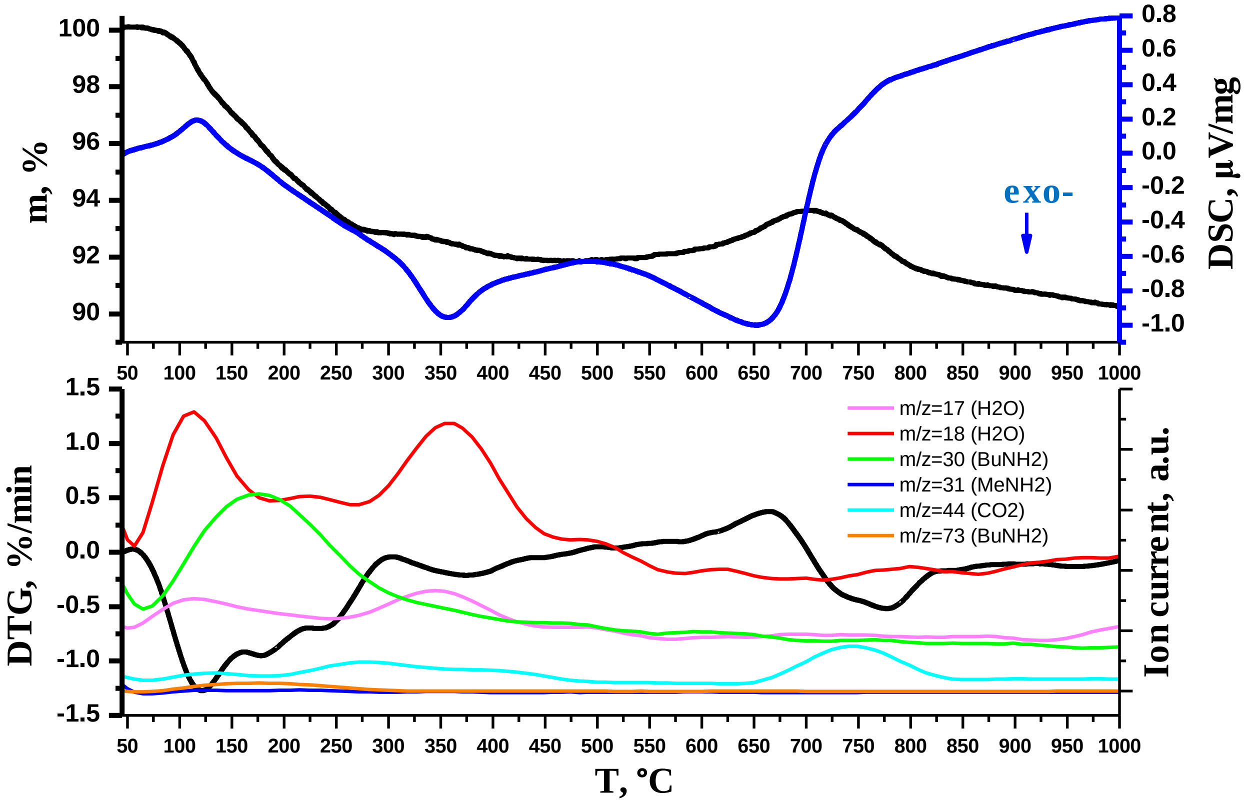

Figure S8. STA-MS data for *n*-butylamine hybrid $HLT_3 \times BuNH_2$

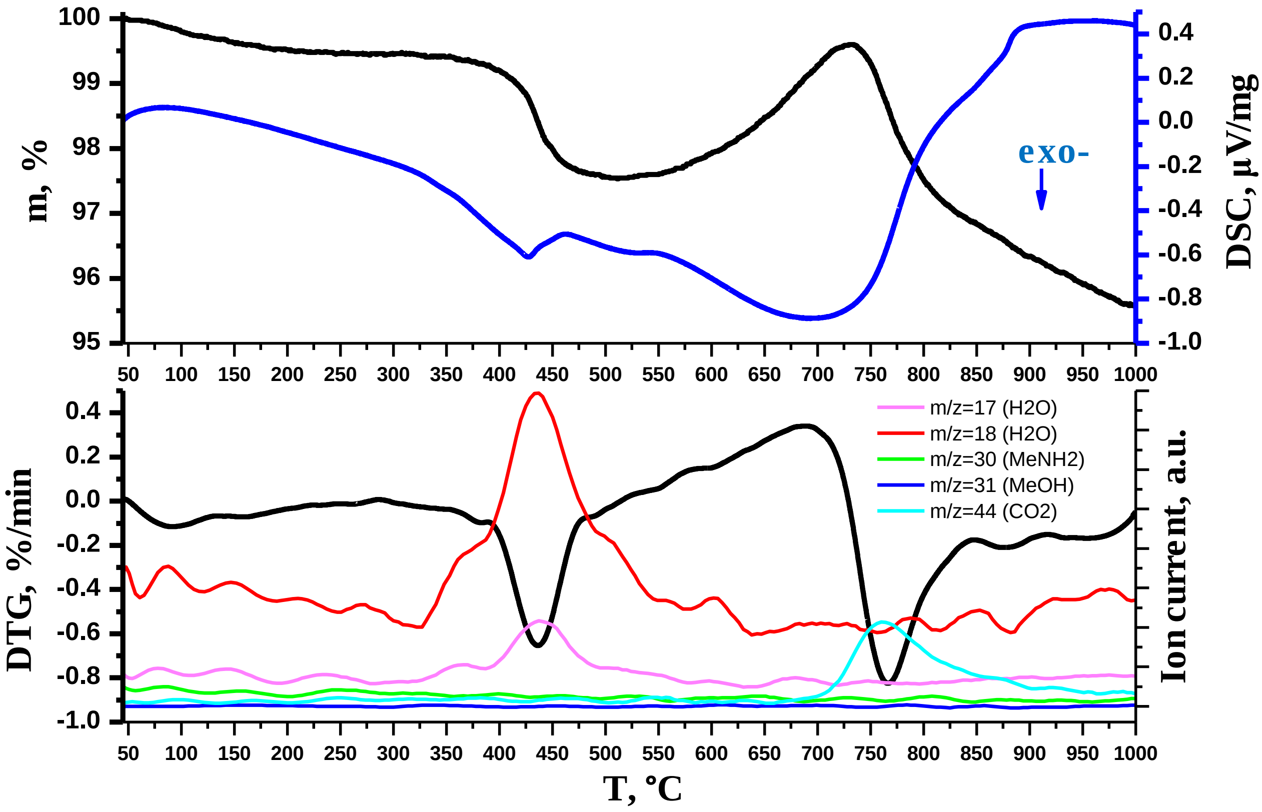

Figure S9. STA-MS data for methanolic hybrid HLT_3×MeOH

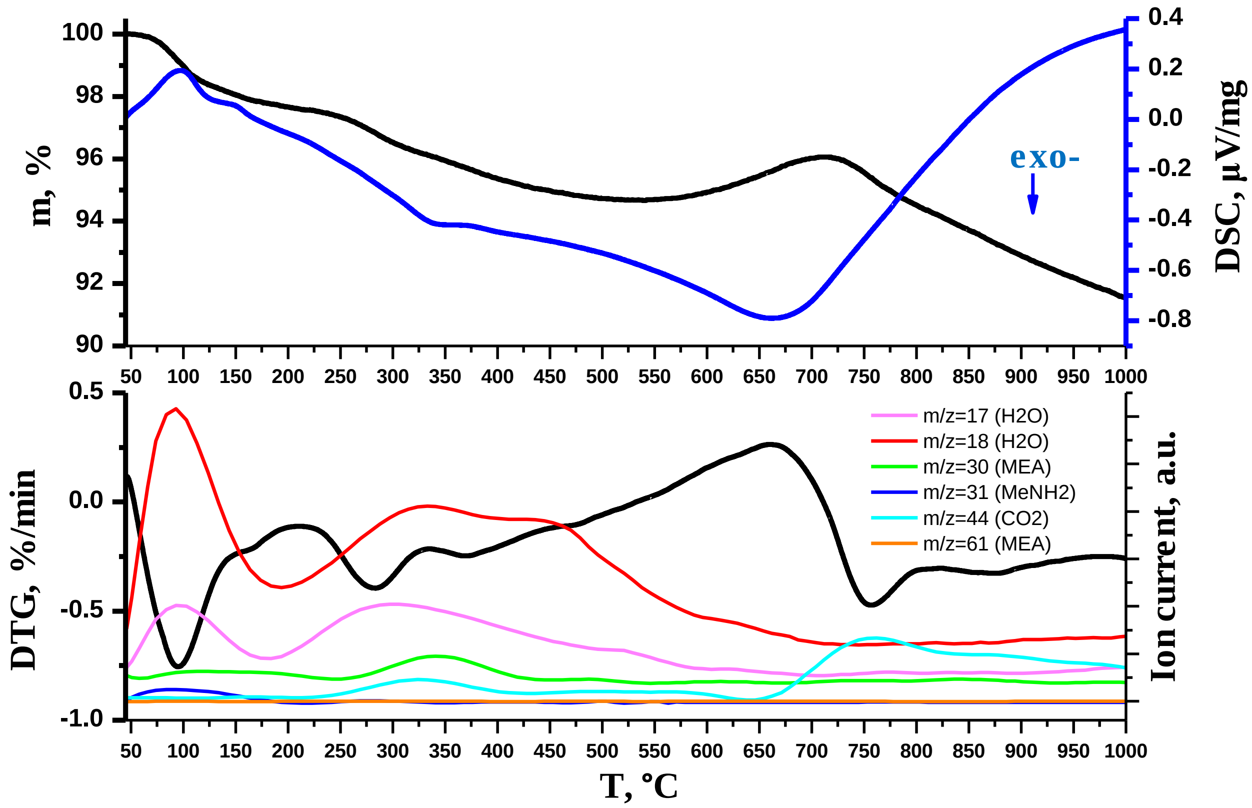

Figure S10. STA-MS data for monoethanolamine hybrid HLT_3×MEA
